# Genomic sequencing in clinical trials

**DOI:** 10.1186/1479-5876-9-222

**Published:** 2011-12-30

**Authors:** Karen K Mestan, Leonard Ilkhanoff, Samdeep Mouli, Simon Lin

**Affiliations:** 1 Department of Pediatrics, Division of Neonatology, Northwestern University Feinberg School of Medicine, Chicago, IL, USA; 2 Department of Medicine, Division of Cardiology, Section of Electrophysiology, Northwestern University Feinberg School of Medicine, Chicago, IL, USA; 3 Department of Radiology, Northwestern University Feinberg School of Medicine, Chicago, IL, USA; 4 Biomedical Informatics Research Center, Marshfield Clinic Research Foundation, Marshfield, WI, USA

**Keywords:** Clinical trial, DNA, sequencing, human genome, bioinformatics

## Abstract

Human genome sequencing is the process by which the exact order of nucleic acid base pairs in the 24 human chromosomes is determined. Since the completion of the Human Genome Project in 2003, genomic sequencing is rapidly becoming a major part of our translational research efforts to understand and improve human health and disease. This article reviews the current and future directions of clinical research with respect to genomic sequencing, a technology that is just beginning to find its way into clinical trials both nationally and worldwide. We highlight the currently available types of genomic sequencing platforms, outline the advantages and disadvantages of each, and compare first- and next-generation techniques with respect to capabilities, quality, and cost. We describe the current geographical distributions and types of disease conditions in which these technologies are used, and how next-generation sequencing is strategically being incorporated into new and existing studies. Lastly, recent major breakthroughs and the ongoing challenges of using genomic sequencing in clinical research are discussed.

## Introduction

Human genome sequencing, the process by which the exact order of nucleic acid base pairs in the 24 human chromosomes is determined, was the most significant technical challenge of the Human Genome Project. Completed in 2003, the 13-year project identified 20,000 to 25,000 genes and determined the sequence of the 3 billion chemical base pairs that make up human DNA as well as the regions that control them. Since then, improvements in sequencing speed, reliability, and cost have been the ongoing goals. Hence, genomic sequencing is rapidly becoming a major part of our translational research efforts to understand and improve human health and disease. With the numerous advances in genomic sequencing, there has been a dramatic increase in the number of clinical trials now using this technology to study key disease outcomes [1].

The objective of this review is to familiarize the translational investigator with genomic sequencing technologies as they apply to clinical trials. We describe the currently available types of genomic sequencing platforms, outline the advantages and disadvantages of each, and compare first- and next-generation techniques with respect to capabilities, quality, and cost. To illustrate the recent impact and widespread movement of genomic sequencing into clinical and translational research, we provide a summary of the types and distribution of clinical studies that are using genomic sequencing to enhance the understanding of complex pathophysiology and identify important biomarkers of both rare and common diseases. We provide some key examples of clinical trials in which translational researchers are utilizing this technology to better identify and individualize the management of high-risk patients, and to achieve major breakthroughs in drug development.

## Features of First- and Next-Generation Sequencing

So rapid are the advances in genomic sequencing technology that the methods are commonly referred to as first- and next-generation sequencing (NGS). Sanger sequencing, developed in the 1990s, was the earliest method used to sequence human DNA. In fact, it was Sanger technology that was used to sequence the human genome in the Human Genome Project. It is often referred to as "first-generation sequencing" because it revolutionized how a single lab could sequence millions (rather than thousands) of base pairs. Sanger sequencing is a multi-channel capillary approach that allows relatively rapid DNA sequencing. Even though the newer platforms are much faster and cheaper, Sanger sequencing remains in many respects the "gold standard" for smaller validation studies, and remains the only widely used platform that can sequence relatively long sequences of DNA--up to 1,000 nucleotides in length (Table 1).

**Table 1:** Summary of throughput, length, quality, and cost of current versions of genomic sequencing.

Platform	Throughput	Length	Quality	Cost	Applications	Sources of error	Advantages	Disadvantages
**Sanger**	6 Mb/day	1,000 nt	10^-4^-10^-5^	~$500/Mb	Small sample sizes, genomes, SNPs, long haplotypes, low complexity regions, etc.	Polymerase/amplification, low intensities/missing termination variants, contaminant sequences	Longest reads, gold standard for validations	High cost, low throughput

**454/Roche**	750 Mb/day	400 nt	10^-3^-10^-4^	~$20/Mb	Complex genomes, SNPs, structural variation, indexed samples, small RNAs, mRNAs, etc.	Amplification, mixed beads, intensity thresholding, homopolymers, phasing, neighbor interference	Longer reads, easier to assemble	Medium throughput, expensive, indel errors more likely

**Illumina**	5,000 Mb/day	100 nt	10^-2^-10^-3^	~$0.50/Mb	Complex genomes, counting (SAGE, CNV Chip, small RNA), mRNAs, structural variation, bisulfite data, indexing SNPs, etc.	Amplification, mixed clusters/neighbor interference, phasing, base labeling	Lower cost, widely adopted platform, most well-developed bioinformatics efforts	Higher base substitution error rate, shorter reads

SOLiD	5,000 Mb/day	75 nt	10^-2^-10^-3^	~$0.50/Mb	Complex small genomes, counting (SAGE, ChiP, small RNA, CNV), SNPs, mRNAs, structural variation, indexing, etc.	Amplification, mixed beads, phasing, signal decline, neighbor interference	Lower cost, 2-base encoding chemistry, higher per-base accuracy	Shortest read lengths, still an emerging platform

Adapted from Kircher, et al. Bioessays 2010^10^

Several second generation sequencing technologies have emerged over the past ten years, including Roche 454, Illumina Genome Analyzer (GA), and Applied Biosystems (ABI) SOLiD. These platforms are able to generate more sequence and are substantially less expensive than the original Sanger methods (Table 1). They can also handle more complex and smaller genomes, sequence mRNA, copy number variants (CNV) and single nucleotide polymorphisms (SNPs) to account for structural variations (Table 2). In addition to whole genome sequencing, NGS technologies have been successfully used in chromatin immunoprecipitation (ChIP)-sequencing to identify key binding sites of DNA-associated proteins, [2,3] and RNA-sequencing for mammalian and human tissue transcriptomes.[4-6] Due to the cost-effectiveness and versatility of NGS as compared to first-generation sequencing, NGS approaches are poised to emerge as a dominant genomics technology in patient-oriented research. Specifically, there is considerable interest in employing NGS platforms for targeted sequencing of specific candidate genes and sequencing of SNPs identified through gene-association studies. With the declining cost of NGS technology, sequencing of the entire human exome in large numbers of individuals is now feasible and promising [7-9].

**Table 2:** Comparison of first-, second-, and third-generation genomic sequencing.

	First generation	Second generation	Third generation
**Fundamental technology**	Size-separation of specifically end-labeled DNA fragments	Wash-and-scan SBS	Single molecule real time sequencing

**Resolution**	Averaged across many copies of the DNA molecule	Averaged across many copies of the DNA molecule	Single DNA molecule

**Current raw read accuracy**	High	High	Lower

**Current read length**	Moderate (800-1000 bp)	Short (generally much shorter than Sanger sequencing)	> 1000 bp

**Current throughput**	Low	High	High

**Current cost**	High cost per base, Low cost per run	Low cost per base, High cost per run	Low cost per base, High cost per run

**RNA-sequencing method**	cDNA sequencing	cDNA sequencing	Direct RNA sequencing

**Time to result**	Hours	Days	< 1 day

**Sample preparation**	Moderately complex, PCR amplification is not required	Complex, PCR amplification is required	Various

**Data analysis**	Routine	Complex (due to large data volumes & short reads)	Complex

**Primary results**	Base calls with quality values	Base calls with quality values	Base calls with quality values

Adapted from Schadt, et al. Hum Mol Genet 2010^13^

Table 1 summarizes the advantages and disadvantages of Sanger, Roche 454, Illumina, and SOLiD platforms [10]. It is important to note that there is no single platform that is ideal for every application, and therefore, all four of these platforms are still widely used. With the lower cost and more rapid turnover of the Illumina and SOLiD platforms, the capacity for read length is limited. Nevertheless, the rapid adoption of genome sequencing has been fueled by rapid dropping of cost, and this may be the main determining factor in which platforms are used for any given clinical trial. The commercial price of a whole genome sequencing declined from more than $50,000 in 2009 to less than $5,000 in 2011 [7,11]. It is anticipated that full genome sequencing will soon cost less than $1,000 [12]. Translational investigators can anticipate ongoing improvements in the existing technologies, and the emergence of newer approaches to offset cost while improving both accuracy, read length, and turnover [13]. For example, Pacific Biosciences released the first "third generation" sequencer this year, which incorporates novel, single-molecule sequencing techniques and advanced analytics. This system can deliver read lengths of > 1,000 bases on average, with results obtained in less than a day, as compared to the current second-generation turnaround of > 1 weeks [14].

## Genomics in Human Disease: Whole, Exome, and Transcriptome Sequencing

There are currently three widely adopted approaches to sequencing: whole genome, exome, and transcriptome sequencing. The specific approach being used for any given study will determine which NGS platform is used. For example, due to its significant expense, whole genome sequencing, in which the entire genome is sequenced, is an arduous and costly methodology to adopt. However, for collecting large amounts of DNA sequence data from individual human subjects, the more expensive Sanger sequencing is still widely used because of its capacity for longer read lengths [15]. An important limitation, however, is the extremely large sample size required to provide adequate power for data analysis in most whole genome studies. Therefore, more cost-efficient methods are needed and the scope of these studies is often driven by the availability of resources and funding. Whole genome and also exome sequencing (in which only the transcribed regions of the genome are sequenced) both attempt to find polymorphisms that may predict drug outcome or explain mono-genic disorders. At the other end of the spectrum, transcriptome sequencing detects gene expression changes and may be used to identify the effects of a drug on patients (pharmacogenomics). For these types of studies, NGS is now rapidly replacing microarray expression analysis, given the capacity of NGS platforms to sequence more complex and smaller genomes [16].

Recent whole genome studies have used NGS to gain insight into genomic markers of disease. An excellent example using whole genome sequencing was reported by Mardis et al, in which 12 somatic mutations within coding sequences and 52 somatic point mutations in conserved regions of patients with acute myeloid leukemia (AML) were identified [17]. Investigators were able to identify two common genetic variants previously linked with AML and two novel markers, one of which was in a non-coding region which demonstrated regulatory potential. Without sequencing the entire genome, investigators may not have understood the influence of non-coding regions on regulatory function in AML, highlighting a potential benefit of this approach. Other more recent areas with promising breakthroughs using whole-genome sequencing include: the development of targeted chemotherapies for lung adenocarcinoma, single-step capture and sequencing of natural DNA for the detection of *BRCA1 *mutations, and the identification *of MYO1E *mutations in childhood familial focal segmental glomerulosclerosis.[18-20]

Exome sequencing has been used to gain insight and determine genetic abnormalities in congenital defects. This approach has been favored for a number of reasons. First, this technique requires only about 5% as much sequencing as a whole genome. In total, there are about 180,000 exons found in the human genome. It is estimated that the protein coding regions of the human genome constitute about 85% of the disease-causing mutations. A pivotal study reported in 2006 identified a point mutation in Freeman-Sheldon Syndrome [21]. After excluding common variants using HapMap, investigators identified the *MYH3 *gene mutation by sequencing four individuals with the disease. Since then, exome sequencing has been used as a popular approach to identify rare Mendelian disorders [22-24]. These breakthroughs have led to the more widespread use of exome sequencing to study and identify specific mutations in more common complex diseases such as breast cancer, [25] familial lipid disorders, [26,27] Parkinson's Disease, [28,29] and autism spectrum disorder [30,31].

Transcriptome sequencing encompasses experiments including small RNA profiling and discovery, mRNA transcript expression analysis (full-length mRNA, expressed sequence tags and ditags, and allele-specific expression) and the sequencing and analysis of full-length mRNA transcripts. Transcriptome investigation has included the areas of novel gene discovery, gene space identification in novel genomes, assembly of full-length genes, SNPs, and insertion-deletion and splice-variant discovery. Transcriptome sequencing has evolved as a robust technique for evaluating gene expression changes from either healthy individuals who develop disease, or within diseases themselves, such as breast cancer and malignant metastases within the same patient [32]. One application of this technology involved a study of lobular breast carcinoma, in which researchers found 32 somatic non-synonymous coding mutations present in the metastasis, and measured the frequency of these somatic mutations in DNA from the primary tumor of the same patient, which arose 9 years earlier. Five of the 32 mutations (in *ABCB11*, *HAUS3*, *SLC24A4*, *SNX4 *and *PALB2*) were prevalent in the DNA of the primary tumor removed at diagnosis 9 years earlier; six (in *KIF1C*, *USP28*, *MYH8*, *MORC1*, *KIAA1468 *and *RNASEH2A*) were present at lower frequencies (1-13%); 19 were not detected in the primary tumor, and two were undetermined [33]. The combined analysis of genome and transcriptome data revealed two new RNA-editing events that recode the amino acid sequence of *SRP9 *and *COG3*. Taken together, these data show that transcriptome sequencing was useful in identifying single nucleotide mutational heterogeneity, which can be a property of low or intermediate grade primary breast cancers, and that significant evolution can occur with disease progression. Most recently, researchers have used transcriptome sequencing approaches to identify functional microRNA involved in endometriosis, and diagnostic and prognostic signatures from the small non-coding RNA transcriptome in prostate cancer [34-36].

## Distribution of Clinical Trials using Genomic Sequencing

**Figure 1:**
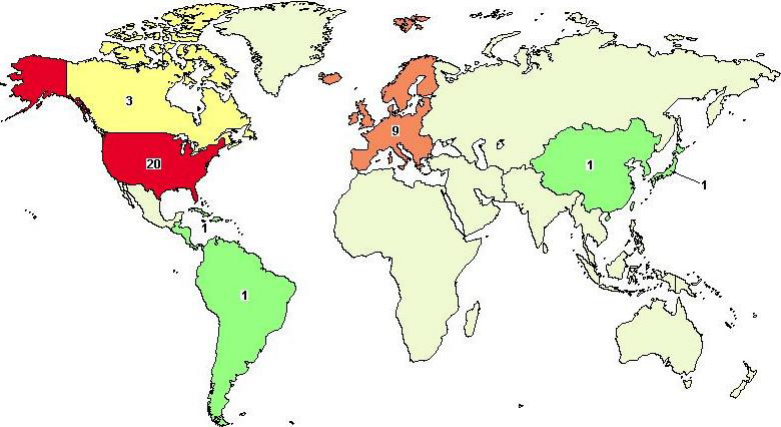
**Worldwide map of clinical trials registered in ClinicalTrials.gov**. Thirty-five studies were found by query of: *genomic sequencing*, based upon a recent search in http://www.clinicaltrials.gov[37] (July, 2011).

**Figure 2:**
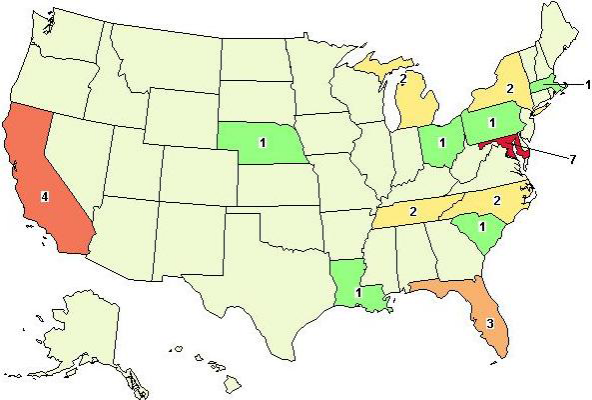
**U.S. distribution of registered clinical trials that disclose the use of genomic sequencing**. In July, 2011, twenty studies reported incorporation of NGS technology. The majority of these studies were being conducted in California (4) and Maryland (7).

Perhaps the best resource for identifying and tracking the growing number of studies now using genomic sequencing is ClinicalTrials.gov [37]. This website is a registry of federally and privately supported clinical trials (experimental and observational) conducted in the United States and around the world. Since 2007, the Food and Drug Administration mandated registration and results reporting for clinical trials of drugs, biologics, and devices (US Public Law 110-85). Based upon our recent search of the website (July 2011) there were 35 registered studies in which "genomic sequencing" was included in the protocol as either a primary or secondary outcome measure. Eighteen of these studies are actively recruiting patients and six have reportedly been completed. Figures 1 and 2 show the worldwide and U.S. distributions of studies involving genomic sequencing that are currently reported on ClinicalTrials.gov. The majority of these are concentrated in the U.S. (20 studies), followed by Europe (9 studies). The highest concentrations of U.S. trials are based in California and Maryland. Clusters of activity appear to be dependent upon the types of research centers located within major academic institutions, but this is not necessarily true for all states.

## Types of Clinical Studies

There is a wide range of studies that are now utilizing genomic sequencing technology. The list of conditions and diseases involved is a clear indicator of the growing interest in understanding the role of genetic predisposition in human disease. According to ClinicalTrials.gov, genomic sequencing technologies have been incorporated into studies on 16 categories of disease conditions. Table 3 lists these categories and examples of associated conditions. There is significant overlap, such that the majority of studies fall under the three most generalized categories involving blood/lymph, cancers/neoplasms, and immune system diseases. The studies most representative of the movement towards genomic sequencing research are those involving cancer biomarker research, including adulthood and childhood leukemias/lymphoma, congenital syndromes and central nervous system disorders, HIV/AIDS research, and associated drug developments.

**Table 3:** Condition categories and diseases studied utilizing genomic sequencing technology.

Condition	Diseases
*Bacterial and Fungal Diseases*	Mycoses, Osteitis, Pelvic Infection, Pelvic Inflammatory Disease, Proteus Infections

*Blood and Lymph Conditions*	Anemia, Blood Coagulation, Burkitt Lymphoma, Hodgkin Disease, Hemorrhagic, Hemoglobinopathies, Leukemias, Lymphomas, Lymphoproliferative Disorders, Multiple Myeloma

*Cancers and Other Neoplasms*	Adenocarcinoma, Neuroblastoma, Nevus, Osteosarcoma, Retinoblastoma, multiple neoplasms, carcinomas, etc.

*Digestive System Diseases*	Digestive System Neoplasms, Duodenal Diseases, Gastroenteritis, Ileal, Jejunal, Stomach Diseases and Neoplasms

*Diseases and Abnormalities at or before Birth*	Multiple congenital anomalies, Cardiovascular Anomalies/Congenital Heart Disease, Inborn Diseases, Hemoglobinopathies, Neurocutaneous Syndromes, Neurofibromatoses

*Ear, Nose, and Throat Diseases*	Deafness, Hearing Disorders, Hearing Loss

*Eye Diseases*	Retinoblastoma

*Gland and Hormone Related Diseases*	Acromegaly, Endocrine Disorders, Dwarfism, Neoplasms, Hyperparathyroidism, Parathyroid and Pituitary Diseases

*Heart and Blood Diseases*	Aortic Valve Stenosis, Arterial Occlusive Diseases, Cardiomyopathies, Coronary Artery Disease, MI

*Immune System Diseases*	AIDS, Lymphomas, Hodgkin Disease, Immunoproliferative Disorders, Leukemias, Myelomas, Mycosis, Macroglobulinemia

*Muscle, Bone, and Cartilage Diseases*	Acromegaly, Bone Diseases, Dwarfism, Congenital Limb Anomalies, Musculoskeletal Abnormalities, Osteitis

*Nervous System Diseases*	ALS, Aphasias, Brain Neoplasms, CNS Diseases, Coma, Communication Disorders, Deafness, Dementia/Delirium, Motor Neuron Diseases, Neurocutaneous Syndromes, Neurodegenerative Diseases, Neurofibromas/NF, Neuromuscular Diseases, Pain, Speech Disorders, Spinal Cord Diseases

*Skin and Connective Tissue Diseases*	Breast Diseases/Neoplasms, Neurocutaneous Syndromes

*Symptoms and General Pathology*	Coma, Communication, Deafness/Delirium, Hearing Disorders, Hemolysis, Inflammation, Ischemia, Neurobehavioral, Pain, Sclerosis, Sepsis/Shock

*Urinary Tract, Sexual Organs, & Pregnancy*	Adnexal Diseases, Renal Cell Carcinoma, Endometritis, Kidney Diseases, Pelvic Inflammatory Disease, Prostatic Neoplasms, Urogenital Neoplasms, Uterine and Urologic Diseases, Wilm's Tumor

*Viral Diseases*	AIDS, Burkitt Lymphoma, HIV Infections

Source: http://www.clinicaltrials.gov (July, 2011)

According to our search, there were six recently completed clinical studies that have proposed genomic sequencing as an outcome measure at the time of this review. In France, an observational study of molecular and metabolic markers in oligodendrogliomas was recently completed with tumor collection from 189 pediatric and adult patients (NCT00213876). Researchers will use these tumor samples to identify diagnostic molecular and metabolic markers that could be used as a signature to characterize benign versus more aggressive tumor histologies. Genomic sequencing and serial analysis of genomic expression results will be correlated to survival and clinical features of oligodendrogliomas, medulloblastomas, and gliomas. A recently completed epidemiological study on the distribution of insulin-like growth factor-1 (IGF-1) deficiency in children with idiopathic short stature will investigate candidate genes and DNA changes that are potentially associated with short stature. DNA regions identified during genome-wide scan will be further mapped at higher resolution using DNA-sequencing (NCT00710307).

Genomic sequencing has also been incorporated as a secondary outcome measure in a few recently completed experimental studies: Investigators in France conducted an open label trial to evaluate the biological effect of Tarceva for patients with epidermoid carcinoma. A frozen tissue bank was generated for genomic sequencing study of tumorous epidermal growth factor receptor (EGF-R) structure and for modification of *in situ *gene expression induction with the drug by RNA microarray technology (NCT00144976). In an international phase II trial of Lapatinib in patients with relapsed or refractory inflammatory breast cancer, researchers will study tumor cell growth and survival by quantitative immunohistochemistry and by direct and genome-wide methods (e.g., direct sequencing and DNA microarray) in tumor tissue collected prior to and following 28 days of lapatinib monotherapy (NCT00105950). In the Dominican Republic, a Phase III randomized, double-blind, placebo-controlled study was completed in 2008 (200 infants) to investigate horizontal transmission of human rotavirus vaccine strain (Rotarix) (NCT00396630). Investigators are using genomic sequencing to analyze mutations in the vaccine strain after transmission.

Thirteen of the 35 studies were designed with genomic sequencing results as a primary outcome measure (Table 4). The most recently registered trial by the Eunice Kennedy Shriver National Institute of Child Health and Human Development (NICHD) will allow for exomic sequencing of participating NICHD patients and their family members. Included in this study are probands that are enrolled in an NICHD clinical protocol for which there is a suspicion of an underlying genetic cause for a disease for which they are being evaluated. Many other studies are capitalizing on up-and-coming NGS technology, including feasibility and pilot studies of solid tumors and leukemias, and whole genome sequencing studies for a wide array of congenital disorders, cardiovascular diseases, hematologic conditions and endocrine disorders. What is common in the diseases being studied is the potential for improved outcome with earlier diagnosis and treatment. Hence, translational researchers are recognizing the potential of genomic sequencing technology as an important diagnostic and screening tool in the clinical setting.

**Table 4:** Active and completed studies using genomic sequencing results as the primary outcome measure (source: http://www.clinicaltrials.gov, July 2011)

Study Title/*Sponsor*	NCT #/ # Enrolled/ Start Date	Condition	Description
Next Generation to Identify Genetic Causes of Disease in Patients Participating in NICHD Clinical Protocols *NICHD*	NCT01375543 100 June 2011	Genetic diseases (pediatric)	Use of DNA samples to conduct exome and genome sequencing

Feasibility Clinical Study of Targeted and Genome-Wide Sequencing *University Health Network, Toronto*	NCT01345513 100 March 2011	Solid Tumors	Targeted and genome-wide sequencing of DNA to enable molecular characterization of tumors.

Biomarkers in Tissue Samples from Patients with High-Risk Wilms Tumor *NCI*	NCT01118078 100 March 2010	Kidney Cancer	Application of array-based methods and NGS to identify candidate molecular targets

Whole Genome Medical Sequencing for Genome Discovery *NHGRI*	NCT01087320 100 Feb 2010	Congenital Syndromes/ Genetic Disorders	Using genomic sequencing to identify genetic causes of disorders that are difficult to identify with existing techniques

Studying DNA in Tumor Tissue Samples from Patients with Localized or Metastatic Osteosarcoma *NCI*	NCT01062438 99 Jan 2010	Sarcoma	Genomic expression profile in osteosarcoma tumor samples using transcriptome sequencing

Genetics of Congenital Heart Disease *Nationwide Children's Hospital*	NCT01192048 1000 Dec 2009	Congenital Heart Disease	Direct sequencing and/or microarray, whole-genome array comparative genomic hybridization (CGH)

Integrated Whole-Genome Analysis of Hematologic Disorders *Stanford University*	NCT01108159 100 Sept 2009	Hematologic Diseases	Whole-genome analysis/high-throughput sequencing using blood, bone marrow and skin biopsy samples

Study of Tissue Samples from Patients with Lymphoma *NCI*	NCT00952809 300 March 2009	Lymphoma; Small Intestinal Cancer	Generation of genome-wide maps of the distribution of nucleosomes and histone modifications as assessed by high throughput sequencing (ChIP-Seq)

Genetics of Endocrine Tumours *Barts & The London NHS Trust*	NCT00461188 150 March 2007	Acromegaly	Tumor samples studied using candidate gene sequencing

DNA Analysis of Tumor Tissue Samples from Patients with Diffuse Brain Stem Glioma *St. Jude Children's Research Hospital*	NCT00899834 30 June 2006	Brain & CNS Tumors	Genome-wide expression of RNA in tumor samples using gene expression profiling. Direct sequencing analysis of tumor DNA

ClinSeq: A Large-Scale Medical Sequencing Clinical Research Pilot Study *NHGRI*	NCT00410241 2000 Dec 2006	Cardiovascular Disease	Sequencing ~ 400 genes related to heart disease

Laboratory Study of Lymphoblasts in Young Patients with High-Risk ALL *NCI*	NCT00896766 150 July 2006	Leukemia	Pilot application of array-based methods and gene re-sequencing to identify candidate molecular targets for ALL

Genome Expression in Lymphoma, Leukemia and Multiple Myeloma *NCI*	NCT00339963 3000 Nov 2001	Lymphoma, Leukemia Multiple Myeloma	Participating centers send samples to the NCI for gene expression profiling, array-based comparative genomic hybridization and cancer gene re-sequencing.

## Largest U.S. Patient Samples

There are a few large-scale epidemiological studies that have begun to incorporate genomic sequencing as a major part of their observations. For example, the largest U.S. sample registered at the time of this review, sponsored by the National Cancer Institute (NCI), has enrolled 3,000 patients to study gene expression of lymphoma, leukemia, and multiple myeloma (NCT00339963). In this trial, DNA sequencing methods are being used to analyze base changes in the genome of the cancer cells. While there are several reports that have described the sequencing of whole genomes from a few patients, the much larger number of cases in this trial will allow researchers to identify biologically relevant patterns in humans [33,38-41]. The National Human Genome Research Institute (NHGRI) has enrolled 2,000 patients to examine genomic sequencing in clinical research on coronary artery disease (ClinSeq, NCT00410241). Researchers will start by sequencing about 400 genes related to heart disease, with the eventual goal of sequencing most or all of participants' genes. The National Institute of Allergy and Infectious Disease (NIAID) has enrolled 1,200 patients to develop diagnostic tests for community acquired pneumonia (CAP) and septic shock (NCT00258869). In this study, advanced bioinformatic, metabolomic, and proteomic approaches will be used with mRNA sequencing to identify protein changes in blood samples that predict outcomes in sepsis and CAP.

## Ethical and Computational Challenges in Translational Medicine

As the cost of NGS technology is becoming less of a limitation, several inherent challenges remain that must not be overlooked. Of utmost relevance to the translational investigator are the ethical, legal and social issues surrounding genomic sequencing. In a recent study conducted by Allen and Foulkes, 30 cancer genome sequencing studies were assessed to evaluate how these issues are being handled across different jurisdictions [42]. While they found a high degree of similarity in how cancer researchers engaged in these studies were protecting participant privacy, there were no consistent means across these studies for re-contacting participants, or for returning results and facilitating participant withdrawal. There was a strong trend towards both using samples for additional, unspecified research and sharing data with other investigators. Given the unique nature of genomic sequencing research, individuals and groups engaging in NGS clinical trials may benefit from human subjects training in these specific areas. However, it is apparent that better-defined consensus standards are still needed both nationally and internationally to prepare the growing number of researchers in this field [43,44].

With the vast amounts of high-quality, complex data now being processed through NGS, an ongoing challenge for translational researchers remains: How do we deal with the computational complexities of analyzing this data? NGS can miss parts of the genome that may be clinically important. It detects mainly small polymorphisms, though it could be used to detect larger copy number variations. Multiple comparison and sample size issues remain an ongoing problem. In general, the computational complexities of properly handling genomic sequencing data have lagged behind the development of the technology. Such challenges may only be addressed through the development of innovative bioinformatic approaches, and through strategic collaboration and knowledgeable study design and implementation by translational investigators. Computerized technological advances are rapidly becoming in high demand [45]. Two promising areas that are being used to bridge the gap between genomics and the bedside are the development of biologically and medically focused text mining algorithms and the integration of the electronic medical record (EMR). Both may speed the process of collection and analysis of structured data. However, these methods require further development and ongoing validation, especially before applying the information in the clinical setting.

## Conclusion

In summary, rapid advances in genomic sequencing have paved the way for the incorporation of NGS into clinical applications. With NGS technology there is much improved cost-effectiveness and more rapid turnover, two critical success factors that are highly appealing to clinical and translational investigators. In the past, studies that implement gene sequencing have been concentrated around major academic institutions, but this is not expected to continue in the future. With more readily available and cost-effective markets now capitalizing on complete human genome sequencing and analysis as an outsourced service, the use of this technology is likely to become more automated, with significant impact on national and international economies [46]. As illustrated in this review, there is an increasing use of genomic sequencing in the U.S. and worldwide, with a wide range of disease conditions now studied that may soon replace microarray approaches in the new era of bioinformatics. Since these technological approaches are highly applicable for both rare Mendelian and well as more complex and common diseases, the future of genomics is promising.

## Abbreviations

AIDS: Acquired Immune Deficiency Syndrome; AML: Acute Myeloid Leukemia; CAP: Community Acquired Pneumonia; ChIP: Chromatin Immunoprecipitation; CNV: Copy Number Variant; DNA: Deoxyribonucleic Acid; EGF-R: Epidermal Growth Factor Receptor; EMR: Electronic Medical Record; GA: Genome Analyzer; HIV: Human Immunodeficiency Virus; IGF: Insulin-like Growth Factor; mRNA: messenger Ribonucleic Acid; NCI: National Cancer Institute; NGS: Next-Generation Sequencing; NHGRI: National Human Genome Research Institute; NIAID: National Institute of Allergy and Infectious Disease; NICHD: National Institute of Child Health and Human Development; RNA: Ribonucleic Acid; SNP: Single Nucleotide Polymorphism.

## Competing interests

The authors declare that they have no competing interests to disclose. Dr. Mestan receives career development funding from NHLBI (K23 HL093302).

## Authors' contributions

KM conducted the research on the Distribution and Types of Clinical Trials, and was primarily responsible for preparing the initial draft of this manuscript for submission, revisions, and for final editing. LI conducted the literature search on Genomics in Human Disease and prepared this section of the manuscript, in addition to critically revising the manuscript for important intellectual content. SM conducted the research on Human Genomic Sequencing and prepared the tables and written sections pertaining to NGS technology. SL is responsible for initiating the topic of this review, overseeing the conception and design, and critically revising for important intellectual content. All authors read and approved the final manuscript.
